# Autoantibody screening in Guillain–Barré syndrome

**DOI:** 10.1186/s12974-021-02301-0

**Published:** 2021-11-01

**Authors:** Cinta Lleixà, Lorena Martín-Aguilar, Elba Pascual-Goñi, Teresa Franco, Marta Caballero, Noemí de Luna, Eduard Gallardo, Xavier Suárez-Calvet, Laura Martínez-Martínez, Jordi Diaz-Manera, Ricard Rojas-García, Elena Cortés-Vicente, Joana Turón, Carlos Casasnovas, Christian Homedes, Gerardo Gutiérrez-Gutiérrez, María Concepción Jimeno-Montero, José Berciano, Maria José Sedano-Tous, Tania García-Sobrino, Julio Pardo-Fernández, Celedonio Márquez-Infante, Iñigo Rojas-Marcos, Ivonne Jericó-Pascual, Eugenia Martínez-Hernández, Germán Morís de la Tassa, Cristina Domínguez-González, Cándido Juárez, Isabel Illa, Luis Querol

**Affiliations:** 1 Neuromuscular Diseases Unit, Department of Neurology, Hospital de la Santa Creu i Sant Pau, Universitat Autònoma de Barcelona, Barcelona, Spain; 2 Immunology Department, Hospital de la Santa Creu i Sant Pau, Universitat Autònoma de Barcelona, Barcelona, Spain; 3 Centro para la Investigación Biomédica en Red en Enfermedades Raras (CIBERER), Madrid, Spain; 4 Centro de Investigación Biomédica en Red en Enfermedades Neurodegenerativas, CIBERNED, Madrid, Spain; 5 Neuromuscular Unit, Department of Neurology, Bellvitge University Hospital, Barcelona, Spain; 6 Neurometabolic Diseases Group, Bellvitge Biomedical Research Institute (IDIBELL), Barcelona, Spain; 7 Department of Neurology, Hospital Universitario Infanta Sofía, Madrid, Spain; 8 Department of Neurology, Hospital Universitario Marqués de Valdecilla (IDIVAL), University of Cantabria, Santander, Spain; 9 Department of Neurology, Hospital Clínico Universitario de Santiago, Santiago, Spain; 10 Department of Neurology, Hospital Universitario Virgen del Rocío, Seville, Spain; 11 Department of Neurology, Hospital Universitario Reina Sofia, Córdoba, Spain; 12 Department of Neurology, Complejo Hospitalario de Navarra, Pamplona, Spain; 13 Department of Neurology, Hospital Clínic de Barcelona, Barcelona, Spain; 14 Department of Neurology, Hospital Universitario Central de Asturias, Oviedo, Spain; 15 Neuromuscular Diseases Unit, Department of Neurology, Research Institute imas12, Hospital Universitario 12 de Octubre, Madrid, Spain

**Keywords:** Guillain–Barré syndrome (GBS), Autoantibodies, Anti-ganglioside, Neurons, Prognosis

## Abstract

**Background:**

Guillain–Barré syndrome (GBS) is an acute inflammatory neuropathy with a heterogeneous presentation. Although some evidences support the role of autoantibodies in its pathogenesis, the target antigens remain unknown in a substantial proportion of GBS patients. The objective of this study is to screen for autoantibodies targeting peripheral nerve components in Guillain–Barré syndrome.

**Methods:**

Autoantibody screening was performed in serum samples from all GBS patients included in the International GBS Outcome study by 11 different Spanish centres. The screening included testing for anti-ganglioside antibodies, anti-nodo/paranodal antibodies, immunocytochemistry on neuroblastoma-derived human motor neurons and murine dorsal root ganglia (DRG) neurons, and immunohistochemistry on monkey peripheral nerve sections. We analysed the staining patterns of patients and controls. The prognostic value of anti-ganglioside antibodies was also analysed.

**Results:**

None of the GBS patients (*n* = 100) reacted against the nodo/paranodal proteins tested, and 61 (61%) were positive for, at least, one anti-ganglioside antibody. GBS sera reacted strongly against DRG neurons more frequently than controls both with IgG (6% vs 0%; *p* = 0.03) and IgM (11% vs 2.2%; *p* = 0.02) immunodetection. No differences were observed in the proportion of patients reacting against neuroblastoma-derived human motor neurons. Reactivity against monkey nerve tissue was frequently detected both in patients and controls, but specific patterns were only detected in GBS patients: IgG from 13 (13%) patients reacted strongly against Schwann cells. Finally, we confirmed that IgG anti-GM1 antibodies are associated with poorer outcomes independently of other known prognostic factors.

**Conclusion:**

Our study confirms that (1) GBS patients display a heterogeneous repertoire of autoantibodies targeting nerve cells and structures; (2) gangliosides are the most frequent antigens in GBS patients and have a prognostic value; (3) further antigen-discovery experiments may elucidate other potential antigens in GBS.

**Supplementary Information:**

The online version contains supplementary material available at 10.1186/s12974-021-02301-0.

## Background

Guillain–Barré syndrome (GBS) is an acute inflammatory neuropathy with a heterogeneous presentation that includes diverse clinical variants [1–3]. Diagnosis is based on clinical criteria; diagnostic biomarkers are not available for most patients [4]. The exact immunopathogenic mechanisms of GBS are relatively unknown, but it is considered a paradigmatic post-infectious autoimmune disease [5]. Diverse mechanisms, including humoral and cellular immune responses, autoantibodies and complement, activated macrophages and lymphocytes, have been implicated in GBS pathogenesis [6, 7].

Anti-ganglioside antibodies are detected in up to half of GBS patients. These autoantibodies arise via microbial molecular mimicry [8] and the association of specific anti-ganglioside antibody reactivities and specific disease variants is well-established in the literature [9, 10], particularly the association of anti-GM1 [11] and GQ1b [12] antibodies with the pure motor and Miller Fisher syndrome (MFS) variants of GBS, respectively. In addition, the presence of antibodies targeting the GM1 [11] or GD1a [13, 14] gangliosides has also been associated with GBS prognosis. Antibodies against nodal and paranodal proteins (neurofascin 140/186 [15] -NF140/186-, neurofascin 155 [16, 17] -NF155-, contactin-associated protein 1 [18, 19] -CASPR1-, and contactin 1 [19] -CNTN1-) have also been described in patients diagnosed of GBS. However, the target antigens remain unknown in a substantial proportion of GBS patients, particularly of the acute inflammatory demyelinating polyradiculoneuropathy (AIDP) variant, the most frequent in patients of European ancestry.

Considering the broad clinical and epidemiological spectrum of GBS, the diverse infectious triggers and the T-cell independent nature of the immune reaction leading to the appearance of autoantibodies [20], we hypothesized that a broad repertoire of autoantibodies targeting diverse nerve components may be causing nerve pathology in GBS. This study aims to (1) screen for autoantibodies against known antigens; (2) screen for antibodies against human and rodent nerve cells and monkey nerve tissue; (3) describe the diversity of staining patterns and (4) perform clinical–immunological correlations, in a well-characterized GBS cohort.

## Materials and methods

### Patients and controls

Serum samples from 100 GBS patients included in the Spanish cohort of the International GBS Outcome study (IGOS) [21] were used in this screening. The IGOS is a multicentre, prospective, observational cohort study that investigates factors that determine and predict the clinical course, subtype and outcome of GBS [22]; including patients fulfilling diagnostic criteria of the National Institute of Neurological Disorders and Stroke (NINDS) [1] or Miller Fisher syndrome (MFS) and other variants of GBS [3, 23]. Patients from the Spanish cohort were enrolled between February 2013 and January 2020. All patients fulfilled diagnostic criteria for GBS and were included within 2 weeks from onset of weakness. Serum samples were aliquoted and stored at − 80 °C until needed. In this study, we used serum samples extracted at baseline. Sixty-two (62%) of the baseline samples analysed were collected before starting treatment.

Clinical variants were defined as sensorimotor, pure motor, pure sensory, Miller Fisher syndrome (MFS) and ataxic. Nerve conduction studies results were classified as acute inflammatory demyelinating polyneuropathy (AIDP), acute motor axonal neuropathy (AMAN), acute motor-sensory axonal neuropathy (AMSAN), equivocal or normal. The outcome of all patients with GBS at 6 months and 1 year from disease onset were assessed using the GBS disability score (GDS), a widely accepted system for evaluating the functional ability of patients [24]. Patients unable to walk independently (≥ 3) at 6 months were defined as having a poor outcome in this study.

Additionally, serum samples from a control group (*n* = 90) including 45 healthy controls and 45 patients with other neuromuscular disorders [23 amyotrophic lateral sclerosis (ALS), 22 Charcot–Marie–Tooth (CMT)] were included.

### Autoantibody screening protocol

Autoantibody screening experiments included anti-ganglioside antibody detection with ELISA, nodo/paranodal (NF155, NF140, NF186, CNTN1 and CNTN1/CASPR1 complex) antibody detection by ELISA and cell-based assays, immunocytochemistry using patient sera on murine dorsal root ganglia (DRG) neurons and neuroblastoma-derived human motor neurons (IgG and IgM) and reactivity pattern assessment by immunohistochemistry on monkey sciatic nerve sections (IgG and IgM).

### Testing for nodo/paranodal antibodies

Autoantibodies against NF140, NF186, NF155, CNTN1 and CASPR1 were tested by ELISA.

Maxisorb 96-well ELISA plates (Thermo Fisher Scientific, NUNC, Denmark) were coated with 1 μg/ml human recombinant CNTN1 protein (Sino Biological Inc., Georgia, USA), 1 μg/ml NF155 protein (Origene, Maryland, USA), 1 μg/ml NF140 protein (Sino Biological), 1 μg/ml NF186 protein (Origene) or 5 μg/ml CASPR1 protein (R&Dsystems, MI, USA) overnight at 4 °C. Wells were blocked with 5% non-fat milk in PBS 0.1% Tween 20 for 1 h, incubated with sera diluted 1/100 in blocking buffer for 1 h, and then incubated with peroxidase conjugated rabbit anti-human IgG secondary antibody (Invitrogen, CA, USA) for 1 h at room temperature. ELISA was developed with tetramethylbenzidine solution (Biolegend, California, USA), and the reaction was stopped with 25% sulfuric acid. Optical density (OD) was measured at 450 nm in a Multiscan ELISA reader. Samples were considered positive by ELISA when they had a ΔOD higher than mean healthy control ΔOD plus two standard deviations.

Cell-based assays were used as previously described [25] as a second confirmatory technique for questionable cases. Briefly, mammalian expression vectors encoding human NF140, NF186, NF155, CNTN1 or CASPR1 were transfected into HEK293 cells using Lipofectamine 2000 (Invitrogen). Cells were then fixed with 4% paraformaldehyde and blocked. ICC experiments were performed using patient’s sera and appropriate primary and secondary antibodies.

### Testing for anti-ganglioside antibodies

Patients’ sera were screened for the presence of anti-ganglioside antibodies using a previously validated ELISA protocol [26] as the general detection method, and thin layer chromatography [27] for confirmatory experiments. Anti-ganglioside antibodies were considered positive at a 1/1000 titre.

### Rat dorsal root ganglia neurons immunocytochemistry

DRG were dissected from E16 rat embryos, dissociated and plated in glass coverslips coated with laminin (Invitrogen) and poly-d-lysine (Sigma, MO, USA). Cells were grown in neurobasal medium (Gibco BRL, NY, USA) supplemented with B27 (Gibco), Glutamax (Gibco) and nerve growth factor (NGF) (Invitrogen). After 24 h, cytosine arabinoside (ARA-C) (Sigma) and fluorouracil (5-FU) (Sigma) were added to the medium to remove fibroblasts. Then, medium was replaced every other day until reaching complete growth and differentiation of DRG neurons.

Live DRG neurons were incubated for 1 h with patients’ sera diluted 1/100 (for IgG experiments) or 1/40 (for IgM experiments) in culture medium at 37 °C. Cells were then fixed for 10 min with 4% PFA and incubated with secondary antibodies. Goat anti-human IgG or IgM AF488 (Molecular probes, Oregon, USA) were used as secondary antibodies at 1/1000 concentration.

Coverslips were mounted with Vectashield with DAPI and fluorescence signal intensity was scored in a 0–3 scale by two independent researchers. Images were obtained with an Olympus BX51 Fluorescence Microscope (Olympus Corporation, Tokyo, Japan).

### Human neuroblastoma-derived neurons immunocytochemistry

SH-SY5Y cells were plated in glass coverslips coated with laminin at 2.5 µg/ml (Invitrogen). Cells were grown in proliferation medium containing DMEM/F12 (1:1), fetal bovine serum (10%), l-glutamine (1%) and Sodium pyruvate (1%). After 24 h, proliferation medium was replaced by differentiation medium containing Neurobasal (Gibco) supplemented with B27 (Gibco), Glutamax (Gibco), nerve growth factor (Invitrogen) and retinoic acid at 10 µM (Sigma). Then, medium was replaced every other day until full differentiation was achieved. On days 5 or 6 of differentiation, cells were fixed for 15 min with paraformaldehyde 4%; and blocked with 5% normal goat serum in PBS; followed by incubation with patients’ sera at 1/40 (for IgM) or 1/100 (for IgG). To observe the correct differentiation of the cells we also incubated them with chicken anti-panNeurofascin mAb (R&Dsystems) at 1/200. Goat anti-chicken IgG AF594 and goat anti-human IgG AF488 or goat anti-human IgM AF488 (Molecular Probes) were used as secondary antibodies at 1/1000 concentration.

Coverslips were mounted with Vectashield with DAPI and fluorescence signal intensity was scored in a 0–3 scale by two independent researchers. Images were obtained with an Olympus BX51 Fluorescence Microscope.

### Peripheral nerve immunohistochemistry

Macaque peripheral nerve tissue slides (Inova Diagnostics, Inc., San Diego, CA) were blocked with 5% normal goat serum in PBS; followed by incubation with patients’ sera at 1:10 (for IgM) or 1:20 (for IgG). Monkey-adsorbed goat anti-human IgG AF488 (Southern Biotech, Alabama, US) or goat anti-human IgM AF488 (Molecular Probes) were used as secondary antibodies at 1/500 concentration. Finally, slides were mounted with Fluoromount medium (Sigma) and examined by two independent observers. Immunostaining patterns were analysed scoring fluorescence signal intensity of each nerve structure in a 0–3 scale. The nerve structures analysed were: nodes or paranodes, myelin from small myelinated fibres, myelin from large myelinated fibres, Schwann cells from unmyelinated fibres, large-fibre axons, and small-fibre axons. Reactivity against other non-nerve structures (fibroblasts, connective tissue, vessels) was not considered in the analysis.

To further study the staining patterns, peripheral nerve tissue slides were coated with mouse anti-human CD56 antibody (Becton Dickinson, New Jersey, USA) at 1:50 to stain non-myelinating Schwann cells (Remak bundles); or with rabbit anti-human S100 antibody (Abcam, Cambridge, UK) at 1:50 to stain myelinating Schwann cells. Goat anti-mouse IgG AF594 (for CD56), and goat anti-rabbit IgG AF594 (for S100) were used as secondary antibodies at 1/500 concentration.

Images were acquired using Leica TSC SP5 confocal microscope.

### Statistical analysis

Results were analysed by GraphPad Prism v8.0 (GraphPad Software). Statistical comparison of proportions among groups was performed using contingency analysis with the application of Chi-square and a two-tailed Fisher’s exact test, accepting an alpha-level < 0.05 for statistical significance. To represent the results and perform hierarchical clustering of the results heatmap diagrams using the Clustvis web tool were performed [28].

To investigate the association between anti-ganglioside antibodies and prognosis a multivariable logistic regression analysis to predict the inability to walk at 6 months and at 1 year of follow-up (GDS ≥ 3) was performed using the STATA software. A stepwise backward regression modelling to select variables independently associated with the outcome was performed first. The variables introduced in our initial multivariable models were selected based on known prognostic factors: age, initial GDS, diarrhoea, AMAN, serum NfL levels (analysed in a previous study with the same cohort) [29], serum anti-GM1 IgG antibodies and serum anti-GD1a IgG antibodies [29–32]. To perform the multivariable analysis patients with MFS were excluded, because our aim was to predict GBS prognosis and MFS is considered a different disease, including different pathophysiology, clinical presentation (it does not present with weakness), treatment (often untreated) and outcome (considered self-limiting and benign). Finally, the ability of the variable “presence of anti-GM1 IgG antibodies” to predict the inability to run at 1 year of follow-up (GDS ≥ 2) was evaluated in our previously reported multivariable logistic regression analysis [29].

Odds-ratios (OR) for the logistic regression analysis were reported with 95% confidence intervals and *p* values.

## Results

### Baseline characteristics

We included 100 participants from 11 Spanish centres participating in the IGOS study. GBS patients had an average of 57.4 years and were predominantly men (57%). 65% of patients presented with the sensorimotor variant, 19% presented with the pure motor GBS variant, 10% with MFS, 5% with the pure sensory variant and 1 patient with the ataxic variant. Regarding nerve conduction studies, 59% of patients were classified as AIDP, 12% as AMAN, 7% as AMSAN, 8% as normal, and 14% as equivocal. Detailed epidemiological features of the cohort were described elsewhere [29].

### Screening for known autoantibodies

None of the GBS patients included in the study reacted against the paranodal and nodal proteins tested (NF155, NF140, NF186, CNTN1 and CASPR1).

Sixty-one patients tested positive for, at least, one anti-ganglioside antibody (GM1, GM2, GM3, GD1b, GD3, aGM1, GT1a, GT1b and GQ1b). Of these, 40 had IgG antibodies, 3 had IgM antibodies, and 18 had antibodies from both isotypes. Detailed anti-ganglioside reactivities are shown in Additional file 1.

Most frequent anti-ganglioside antibodies in our cohort were aGM1, GM1, GD1b and GQ1b. Overall, IgG anti-aGM1 antibodies were detected in 40% of patients; IgG and IgM anti-GM1 antibodies were detected in 27% and 15% of patients, respectively, IgG anti-GD1b antibodies in 30%, and IgG anti-GQ1b antibodies in 21% patients.

### Antibodies targeting peripheral nerve neurons

ICC experiments with primary cultures of rat DRG neurons and human motor neurons derived from a neuroblastoma cell line were used to identify novel IgG and IgM reactivities against peripheral nerve neurons. The screening was performed in 100 serum samples from GBS patients and 90 serum samples from a control group (including healthy controls and patients with other neuromuscular diseases). ICC results were grouped in three separate categories: moderate-to-strong positives (including scores 2 and 3), all positives (including scores 1, 2 and 3), and negatives (score 0). Detailed results are shown in Additional file 2, Fig. 1 and Table 1.

**Fig. 1:**
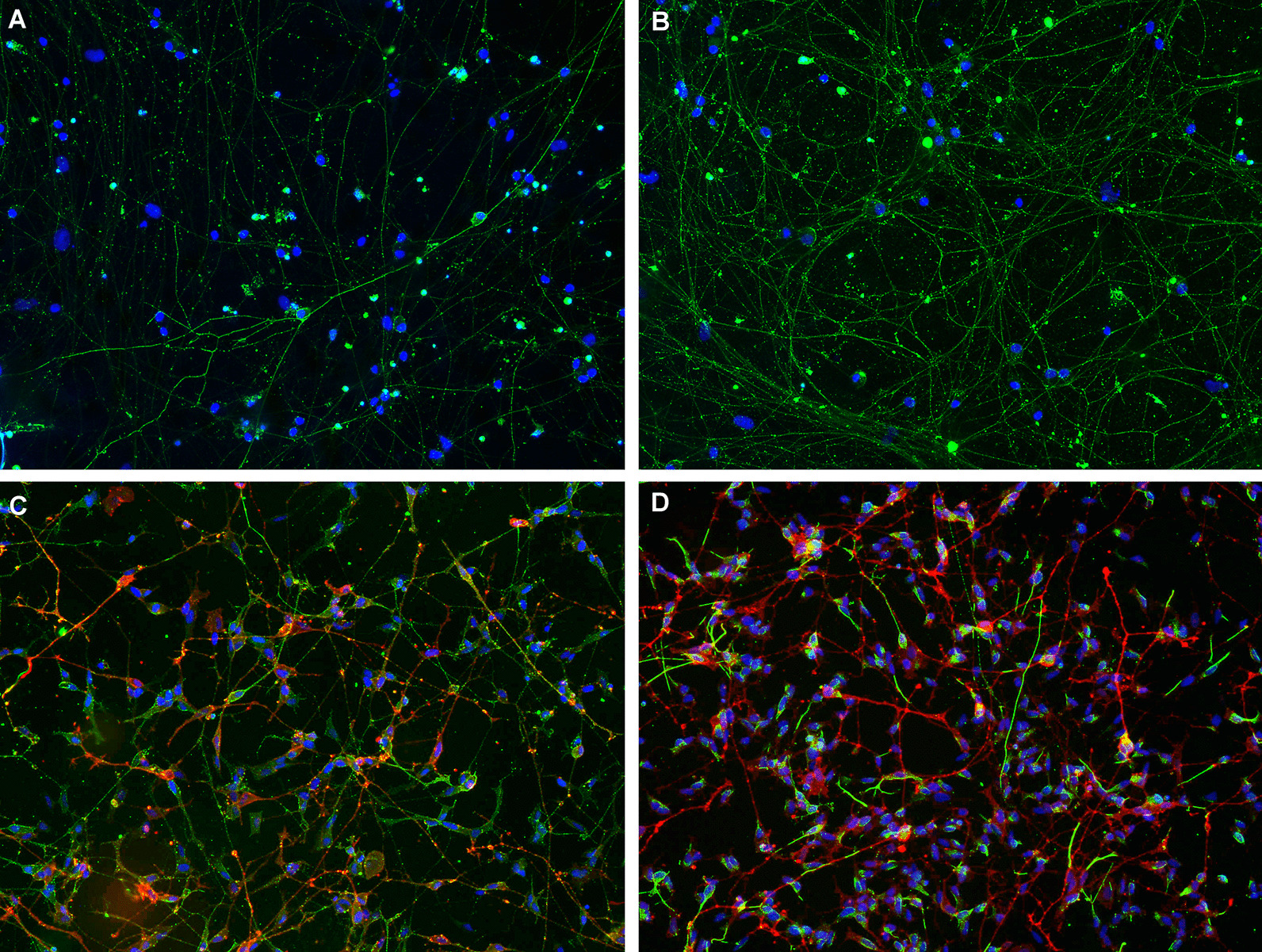
Reactivity against rat DRG neurons and human neuroblastoma-derived neurons. DRG neurons (**A**, **B**) stained with a GBS patient’s serum reacting moderately (score 2) in IgG (**A**), and a GBS patient’s serum reacting strongly (score 3) in IgM (**B**). Human neuroblastoma-derived neurons (**C**, **D**) stained in red with anti-panNeurofascin mAb, and in green with a GBS patient’s serum reacting moderately (score 2) in IgG (**C**) and a GBS patient’s serum reacting strongly (score 3) in IgM (**D**)

**Table 1:** Statistical analysis of DRG and neuroblastoma neurons ICC, and monkey peripheral nerve IHC

	GBS patients (*n* = 100)		Controls (*n* = 90/56)		*p* value	
	Any reactivity	Strong reactivity	Any reactivity	Strong reactivity	Any reactivity	Strong reactivity
Neuroblastoma neurons IgG	11 (11%)	2 (2%)	10/90 (11.1%)	0/90 (0.0%)	> 0.999 (ns)	0.499 (ns)
Neuroblastoma neurons IgM	28 (28%)	8 (8%)	11/90 (12.2%)	2/90 (2.2%)	0.011 (*)	0.105 (ns)
DRG neurons IgG	32 (32%)	6 (6%)	6/90 (6.7%)	0/90 (0.0%)	< 0.0001 (***)	0.030 (*)
DRG neurons IgM	34 (34%)	11 (11%)	22/90 (24.4%)	2/90 (2.2%)	0.156 (ns)	0.020 (*)
Monkey peripheral nerve IgG	56 (56%)	17 (17%)	30/56 (53.6%)	3/56 (5.4%)	0.8669 (ns)	0.0455 (*)
Monkey peripheral nerve IgM	44 (44%)	10 (10%)	20/56 (35.7%)	6/56 (10.7%)	0.3964 (ns)	> 0.999 (ns)

Comparison between GBS patients and controls. *Strong reactivity* includes scores 2 and 3, and *any reactivity* includes scores 1, 2 and 3. Fluorescence intensity scores were analysed using contingency analysis with the application of a Fisher’s exact test, accepting an alpha-level of < 0.05 to determine significance

Overall, 22 (22%) GBS patients reacted moderately or strongly against DRG or neuroblastoma neurons, whereas 4 (4.4%) controls reacted only moderately. These differences were statistically significant (*p* = 0.0005).

Antibodies against DRG neurons appeared significantly more frequently in GBS patients than in controls (32% vs 6.7%, *p* < 0.0001) taking all positive tests in account; the same happened if only moderate and strong positives were considered, both in IgG (6% vs 0%, *p* = 0.03) and IgM experiments (11% vs 2.2%, *p* = 0.02).

In neuroblastoma-derived neuron ICC experiments 28 (28%) samples from the GBS group showed IgM autoantibodies; of these 8 (8%) showed moderate or strong reactivity. These proportions were significantly higher than in the control group (12.2% and 2.2%, respectively; *p* = 0.011). Differences in autoantibody proportions between GBS patients and controls were not observed in neuroblastoma-derived neuron experiments when assessing IgG antibodies.

### Antibodies targeting peripheral nerve tissue

We analysed the full GBS cohort and 56 controls.

We analysed the staining intensity of six different structures within the nerve, including nodes or paranodes, myelin from small myelinated fibres, myelin from large myelinated fibres, Schwann cells from unmyelinated fibres, large-fibre axons, and small-fibre axons. Staining patterns can be found in Additional file 3.

IgG and IgM reactivity against nerve tissue was frequently detected in GBS patients and controls. Overall, about 70% of GBS patients and controls sera bound to nerve structures. IgG and IgM from GBS patients reacted moderately in 17 (17%) and strongly in 10 (10%) against monkey nerve structures. In the control group IgG and IgM reacted moderately in 8 (14.3%) and strongly in 1 (1.8%) against monkey nerve structures. The difference between the amount of GBS patients and controls reacting moderately or strongly against monkey peripheral nerve was statistically significant (*p* = 0.0455) only for IgG autoantibodies (Table 1).

**Fig. 2:**
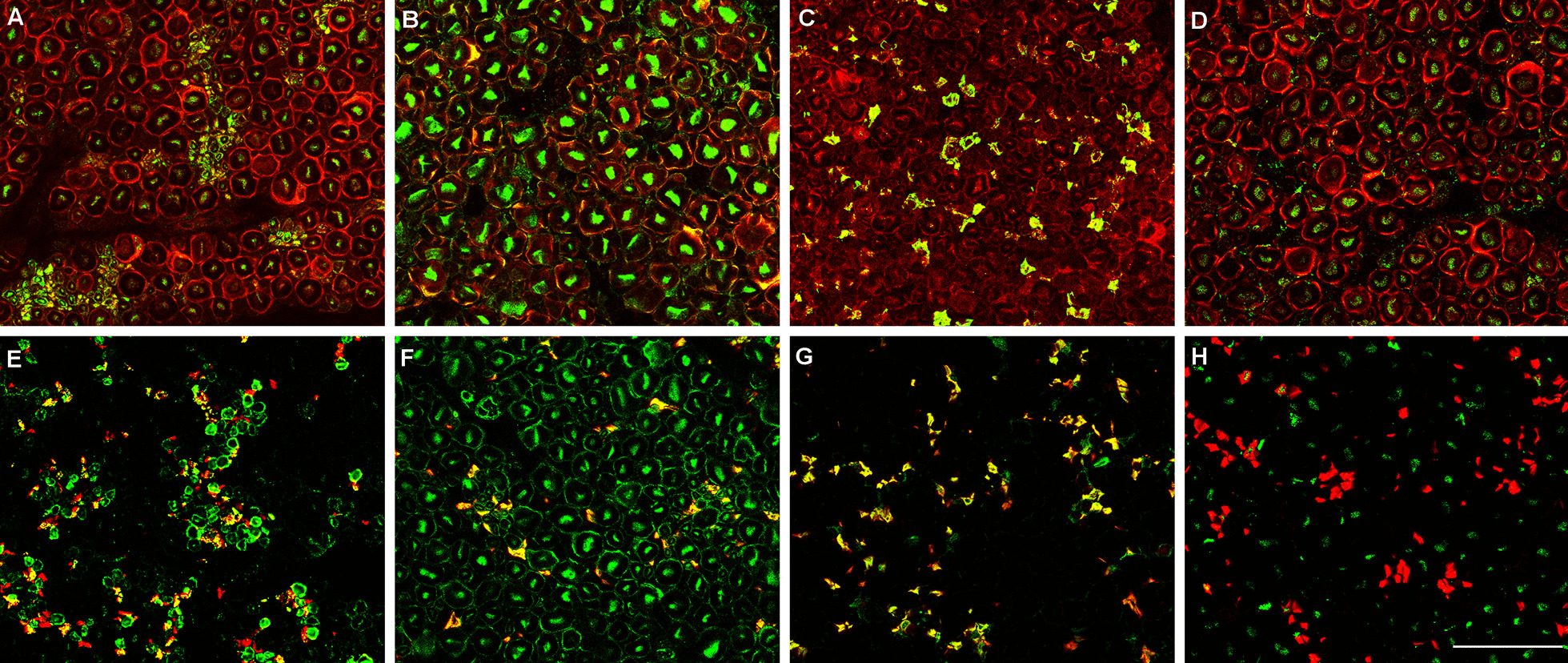
Reactivity against Schwann cells in peripheral nerve sections. Macaque peripheral nerve sections stained in red with S100 (**A–D**) or CD56 (NCAM) monoclonal antibody (**E**–**H**), and in green with GBS patient’s sera reacting against myelin from small myelinated fibres (**A**, **E**), myelin from large myelinated fibres (**B**, **F**), and non-myelinating Schwann cells (**C**, **G**). **D** and **H** are stained with sera from negative controls

Differences in IHC patterns of reactivity from GBS patients and controls were not statistically significant for any of the structures analysed (Additional file 4). Nonetheless, some specific reactivity patterns were only detected in GBS patients and not in controls (Fig. 2). Eight (8%) GBS patients’ IgG reacted strongly against myelin, whereas only 2 controls showed weak reactivity against this structure. Moreover, we observed that 13 (13%) GBS patients’ IgG had a strong reactivity against Schwann cells (myelinating and non-myelinating) while only one of the controls (1.8%) showed strong reactivity against Schwann cells (this difference is statistically significant; *p* = 0.0192).

### Combined autoantibody screening analysis

We also analysed if GBS patients with or without anti-ganglioside antibodies differed in the reactivity patterns in the peripheral nerve cell and tissue autoantibody screening experiments. No differences were found between those two groups (Additional file 4), suggesting that the heterogeneity of the autoantibody repertoire appears even when a specific antigen is found.

**Fig. 3:**
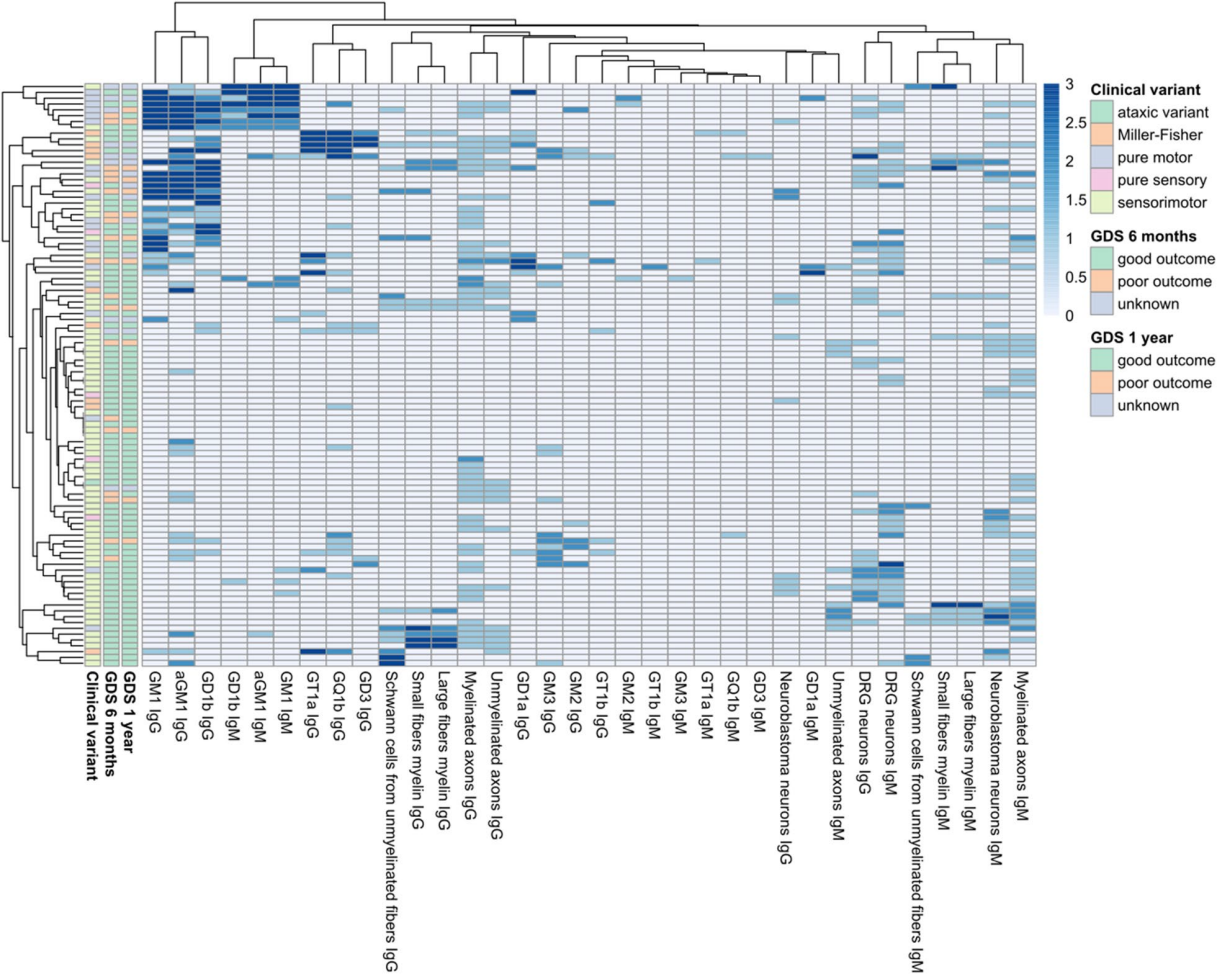
Heatmap showing all the screening performed in GBS patients. Patients and reactivities against anti-ganglioside antibodies, neuroblastoma-derived human motor neurons, murine dorsal root ganglia neurons and monkey peripheral nerve tissue, are ordered according to Euclidean clustering. Each row represents one GBS patient. The score of the anti-ganglioside titre is indicated by the colour of the square (0 =  < 1/1000, 1 = 1/1000–1/2500, 2 = 1/2500–1/12500, 3 =  > 1/12500). The score of staining intensity in the other structures is indicated by the colour of the square (0 = negative, 1 = mild positive, 2 = moderate positive, 3 = strong positive). Columns in the left contain information related to the clinical variant and the outcome at 6 months and at 1 year of follow-up

We used a heatmap graph to represent all the autoantibody screening results performed in our GBS cohort (Fig. 3) [28]. This graph provides visual representation of the heterogeneity of the autoantibody repertoire in GBS sera.

### Clinical correlations

Among patients with Miller Fisher syndrome, 8/10 (80%) had IgG anti-GQ1b antibodies, whereas in the rest of GBS patients only 14.4% (13/90) had these antibodies, usually in combination with other reactivities. IgG anti-GM1 antibodies were more frequently detected in patients with the pure motor variant than in those with other clinical variants [13/19 (68.4%) vs 14/81 (17.3%)]; and in patients classified electrophysiologically as AMAN than in the rest of GBS patients (83.3% vs 19.3%). All these differences were statistically significant (*p* < 0.0001).

When we analysed the general clinical characteristics of the subgroup of patients with strong IgG reactivity against Schwann cells (*n* = 13), we did not observe any specific pattern that could distinguish them from the rest of the cohort. Ten (76.9%) of these patients presented with the sensorimotor clinical variant, whereas in the general cohort 65% presented this variant; and the proportions of nerve conduction studies subgroups were similar to those found in the general cohort (53.8% vs 59% of AIDP electrophysiological variant). Regarding the outcome, the percentage of patients having a good outcome at 6 months and 1 year are similar in the two groups (about 75%).

In the subgroup of patients with IgG or IgM reactivity against DRG neurons (*n* = 14), we did not find clinical differences with the whole GBS cohort. Briefly, 71.4% of patients staining strongly DRG neurons were classified as AIDP, and 64.3% presented with the sensorimotor clinical variant.

We did not detect any difference in peripheral nerve cell and tissue reactivity patterns or frequencies between the samples collected before starting the treatment (62%) and those collected after the treatment (38%). We neither observed any correlation of any of the reactivity patterns with other clinical characteristics as pain, ataxia, or the presence of antecedent infection.

### Prognostic value of anti-ganglioside antibodies

First, we conducted a univariate analysis to select variables that were associated with the outcome. Patients with serum IgG anti-GM1 antibodies presented poorer outcomes than patients without the antibodies at 6 months [38.1% vs 16.1% (*p* = 0.04)], and 1 year [35.3% vs 9.7% (*p* = 0.014)]. Anti-GD1a IgG antibodies were not associated with prognosis (Table 2). For the multivariate analysis, we included GM1 IgG, serum NfL levels, diarrhoea, age, and initial GDS.

**Table 2:** Association between baseline anti-GM1 and anti-GD1a antibodies and prognostic

Univariate and Multivariable logistic analysis for inability to walk independently at 6 months					
Variable	OR		95% CI		*p*
*Univariate analysis*					
GM1 IgG	3.2		1.05–9.71		0.04
GD1a IgG	0.48		0.05–4.24		0.515
logNFL	3.22		1.46–7.14		0.004
AMAN	2.97		0.73–12.07		0.127
Diarrhoea	3.64		1.21–10.91		0.021
Age	1.05		1.01–1.10		0.005
Initial GDS	3.15		1.38–7.18		0.006
*Multivariable logistic analysis*					
logNfL	3.13		1.27–7.67		0.012
Age	1.05		1.01–1.10		0.013

Univariate and Multivariable logistic analysis for inability to walk independently at 1 year			
Variable	OR	95% CI	*p*
*Univariate analysis*			
GM1 IgG	5.09	1.38–18.73	0.014
GD1a IgG	0.92	0.10–8.44	0.944
logNFL	3.11	1.27–7.55	0.012
AMAN	1.475	0.27–7.97	0.652
Diarrhoea	2.7	0.74–9.82	0.131
Age	1.06	1.01–1.11	0.014
Initial GDS	2.79	1.12–6.93	0.027
*Multivariable logistic analysis*			
GM1 IgG	6.98	1.60–30.36	0.01
Age	1.07	1.01–1.12	0.01

Univariate and Multivariable logistic analysis for inability to run independently at 1 year						
Variable	OR		95% CI		*p*	
*Univariate analysis*						
GM1 IgG	4.16		1.39–12.49		0.011	
GD1a IgG	0.72		0.13–3.99		0.710	
logNFL	4.37		1.95–9.78		< 0.0001	
AMAN	6.53		1.57–27.17		0.01	
Diarrhoea	2.33		0.83–6.57		0.109	
Age	1.04		1.01–1.07		0.023	
Initial GDS	1.81		1.08–3.04		0.025	
*Multivariable logistic analysis*						
Age	1.04		1.01–1.08		0.022	
AMAN	6.19		1.01–38.02		0.049	
logNfL	3.17		1.34–7.50		0.009	

*logNfL* log-transformed neurofilament light chain; *AMAN* acute motor axonal neuropathy; *GDS* Guillain–Barré Syndrome Disability Score

We observed that having anti-GM1 IgG antibodies at baseline was independently associated with the inability to walk at 1 year of follow-up, after a backward stepwise selection modelling (OR 6.98, 95% CI 1.6–30.36; *p* = 0.01). However, the presence of anti-GM1 IgG antibodies was not independently associated with having a poor outcome at 6 months (Table 2).

To analyse if anti-GM1 titres were associated with the GBS disability score, we performed a linear regression. We did not observe a positive correlation between antibody titres and disability at 6 months and at 1 year.

Finally, when we included the presence of anti-GM1 antibodies in our previously reported prognostic study [29], we observed that having anti-GM1 IgG antibodies at baseline was associated with the inability to run at 1 year, but this association was not independent from the other known prognostic factors and sNfL, age and AMAN remained in the model as independent factors associated with residual disability at 1 year.

## Discussion

Our work describes a comprehensive autoantibody screening that provides experimental evidence of the heterogeneity of the autoantibody repertoire in patients fulfilling GBS diagnostic criteria.

Our study shows that GBS patients have a heterogeneous repertoire of autoantibodies targeting nerve cells and structures. Except for patients with anti-ganglioside antibodies and a minor subset of patients with antibodies targeting Schwann cells and the myelin sheath, this repertoire varies in frequency and intensity of staining, but it is not qualitatively different from controls. Antibodies targeting peripheral nerve cells of both IgG and IgM isotypes are significantly more frequent in patients than in controls, but no clear differences are seen when antibodies are tested using immunohistochemistry on monkey nerve preparations. Considering that whole nerve monkey preparations likely display protein antigens in a conformation that is phylogenetically closer to that of human nerves, this may imply that autoantibodies targeting nerve structures are present in normal human repertoire at lower titers, that they arise as a natural epiphenomenon of a T-cell mediated damage and are not pathogenic, or that other autoantibodies, targeting different types of molecules (such as lipids or glycans), for which our techniques are not optimized, are yet to be discovered.

Whether these autoantibodies arise from a process of molecular mimicry, or from an unspecific and polyclonal activation of pre-existing B cells, remains unclear. The general absence of common patterns suggests the latter, but the well-established molecular mimicry process described in anti-ganglioside-associated GBS supports the former. In anti-GM1-associated GBS the sequence of pathogenic events includes an immune response to an infection leading to the appearance of antibodies cross-reacting with peripheral nerve and nerve root gangliosides and triggering post-infectious inflammation [33]. Interestingly, in this screening we did not find clear differences in the reactivity patterns between GBS patients with or without anti-ganglioside antibodies, but we observed in both groups a higher amount of patients staining nerve structures than in controls. These findings suggest that the immune response in GBS is not restricted to the production of anti-ganglioside antibodies, but it is also targeting other peripheral nerve structures. This observation may either reflect the presence of a polyclonal, not antigen-driven, reactivation of a pre-existing repertoire that, in some patients, includes gangliosides, or the concomitant activation (by epitope spreading or bystander activation) of unspecific B cells in addition to the ganglioside-driven antigen-specific response.

Previous studies in other inflammatory neuropathies such as chronic inflammatory demyelinating polyneuropathy (CIDP), showed that frequencies of reactivity against DRG neurons in CIDP patients did not differ from healthy controls [34], in contrast with our results (shown in Table 2). GBS and CIDP are similar diseases both clinically and electrophysiologically, so this difference supports the idea that a heterogeneous autoantibody response against multiple nerve antigens arises in GBS while this does not happen in chronic inflammatory and demyelinating neuropathies in which a specific, antigen-driven autoantibody response arises, as the recent discovery of the nodo-paranodal antibodies supports [35]. Differences in severity of these two diseases may also account for this observation.

We observed that 13% of GBS patients showed strong IgG reactivity against Schwann cells of monkey peripheral nerve. This observation is in agreement with previous findings: Kwa et al. observed that 24% of GBS patients had IgG antibodies against non-myelinating human Schwann cells [36], and Vallat et al. also detected that a significant percentage of CIDP and GBS patients (about 25%) presented with IgG or IgM reactivity against myelin and that the staining patterns on Schwann cells were diverse, suggesting that diverse myelin antigens are being recognized by the autoantibodies [37].

Our study also confirms, in a well-characterized GBS cohort, that gangliosides are the most frequent specific antigens in GBS patients and that they associate to specific disease variants. The value of testing anti-ganglioside antibodies in the GBS routine clinical care is controversial, but it is clear that some antibodies are associated with specific clinical phenotypes [38]. IgG anti-GQ1b antibody is a diagnostic marker and a pathogenic antibody in MFS, and is often cross-reactive with GT1a [9]. Moreover, IgG anti-GM1 antibodies associate with the pure motor (clinical) and AMAN (electrophysiological) variants. Our results, with 80% of MFS patients having anti-GQ1b antibodies and 68.4% of pure motor patients having anti-GM1 antibodies at baseline, confirm these associations. However, our study lacks power to find other potential associations previously described (anti-GD1b with acute ataxic neuropathy, anti-GT1a and pharyngo-cervico-brachial variant) [39, 40] that will need to be confirmed in even larger cohorts. Likewise, the clinical relevance of antibodies targeting other structures (neurons, peripheral nerve tissue…) is unclear, since their association with GBS is not completely specific or the number of patients with each particular reactivity is too low to draw any conclusions.

Some studies have reported a correlation between IgG anti-GM1 and anti-GD1a antibodies with a poor outcome in GBS patients [13, 14, 33, 41]. In our cohort, IgG anti-GD1a antibodies did not associate to a poor outcome of the disease [13]. However, our data confirm that IgG anti-GM1 antibody is an independent prognostic factor that associates with poor prognosis at 1 year, supporting that it may be a marker for long-term axonal damage. Whether the presence of complement-fixing anti-GM1 antibodies is the driver of this long-term disability, an important therapeutic question (that would enable the use of complement inhibitors in these patients), remains to be elucidated.

Although in this study we analysed the prognostic value of anti-ganglioside antibodies using the traditional outcome measures: inability to walk (GDS ≥ 3) at 6 months and at 1 year, we have recently used the inability to run (GDS ≥ 2) as a measure of the presence of long-term residual disability. In this recent study we showed that high baseline sNfL were independently associated with inability to run at 1 year [29]. In agreement with these findings, we observed that including in the model the variable “presence of serum IgG anti-GM1 antibodies”, sNfL levels remained as an independent prognostic factor, whereas anti-GM1 antibodies did not. These results confirm that sNfL levels are a prognostic factor that informs better on axon status and, consequently, on long-lasting disability.

It is interesting to note that we did not find any patient with anti-nodal/paranodal antibodies (CNTN1, NF140, NF186, NF155 and CASPR1) in our GBS cohort. Although previous studies from other authors have found some GBS positive patients in their cohorts [14–18], and case-reports and series describe the association of anti-nodal/paranodal antibodies with aggressive inflammatory neuropathies frequently misdiagnosed as GBS, these antibodies are rare and we cannot rule out the possibility that they are present in other selected patients that our cohort failed to capture.

One of the limitations of our study is the number of patients and controls included. We have small groups of patients with similar staining patterns in which it is difficult to establish clear clinical-immunological correlations. Nevertheless, this is the first large prospective study assessing the autoantibody repertoire against peripheral nerve structures in GBS patients and antigen-identification experiments will follow in those patients showing specific staining patterns that are absent in controls.

The existence of clear subgroups associated with anti-ganglioside antibodies, in contrast with the diversity in the new reactivities analysed, suggests that this apparent heterogeneity may be also due to technical caveats, because our study protocol is optimized for proteins and not for lipids or glycans. Moreover, other, not properly controlled factors, could have influenced heterogeneity in staining patterns (treatment, comorbidities…), and will need to be assessed in larger cohorts.

## Conclusions

In conclusion, our study highlights the heterogeneity of the profile of autoantibodies targeting peripheral nerve structures, confirms gangliosides as the most frequent target antigens in the GBS autoantibody repertoire and their prognostic value in long-term GBS prognosis, and identifies small subsets of GBS patients with specific staining patterns in which further antigen-identification experiments could demonstrate novel and clinically relevant autoantibody reactivities in the future.

## Supplementary Information

**Additional file 1: Figure 1.** Heatmap showing anti-ganglioside antibodies in the GBS cohort. Patients and reactivities against anti-ganglioside antibodies are ordered according to Euclidean clustering. Each row represents one GBS patient. The score of the anti-ganglioside titre is indicated by the colour of the square (0 =  < 1/1000, 1 = 1/1000–1/2500, 2 = 1/2500–1/12500, 3 =  > 1/12500). Columns in the left contain information related to the clinical variant and the outcome at 6 months and at 1 year of follow-up.

**Additional file 2: Figure 2.** Staining paterns analized in ICC over rat DRG neurons and human neuroblastoma-derived neurons. DRG neurons (**A**–**H**) or neuroblastoma neurons (**I**–**P**) were stained with control or patient’s sera in green. Signal intensity of IgG (**A**–**D**, **I**–**L**) or IgM (**E**–**H**, **M**–**P**) reactivity was scored in a 0–3 scale (0 = negative, 1 = mild positive, 2 = moderate positive, 3 = strong positive). Neuroblastoma neurons were counterstained in red with anti-panNeurofascin mAb.

**Additional file 3: Figure 3.** Staining paterns analized in IHC over monkey peripheral nerve. Macaque peripheral nerve transverse sections stained with CNTN1 positive CIDP patient’s serum reacting against paranodes (**A**), small fiber axons (**B**), non-myelinating Schwann cells (**C**), myelin from small myelinated fibers (**D**), large fiber axons (**E**), and myelin from large myelinated fibers (**F**).

**Additional file 4: Table 1.** Statistical analysis of structures observed in IHC over monkey peripheral nerve. **Table 2.** Statistical comparison between GBS patients with and without anti-ganglioside antibodies.

## Data Availability

The datasets used and/or analysed during the current study are available from the corresponding author on reasonable request.

## Abbreviations

GBS: Guillain–Barré syndrome
IGOS: International GBS Outcome Study
DRG: Dorsal-root ganglia
MFS: Miller Fisher syndrome
AIDP: Acute inflammatory demyelinating polyneuropathy
AMAN: Acute motor axonal neuropathy
AMSAN: Acute motor–sensory axonal neuropathy
GDS: GBS disability score
ALS: Amyotrophic lateral sclerosis
CMT: Charcot–Marie–Tooth
NF155: Neurofascin-155
NF140: Neurofascin-140
NF186: Neurofascin-186
CNTN1: Contactin-1
CASPR1: Contactin-associated protein 1
PFA: Paraformaldehyde
NfL: Neurofilament light chain
OR: Odds-ratio
CIDP: Chronic inflammatory demyelinating polyneuropathy
