# Supplementary figures and images for: Autoantibody screening in Guillain–Barré syndrome
Source: J Neuroinflammation. 2021 Nov 1;18:251. doi: 10.1186/s12974-021-02301-0 (PMC8559393; doi:10.1186/s12974-021-02301-0)

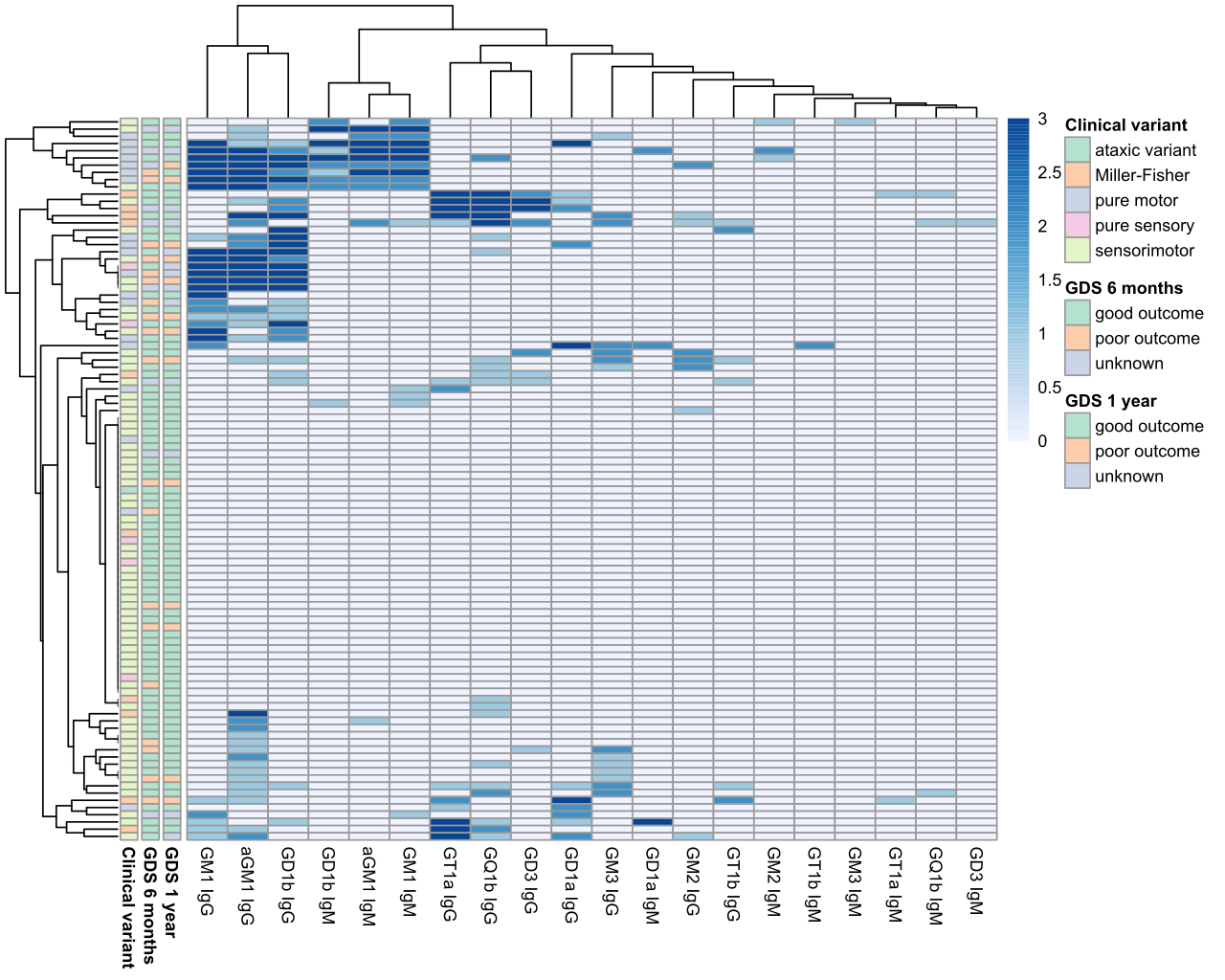

Supplement: Supplementary file 1 — Additional file 1: Figure 1. Heatmap showing anti-ganglioside antibodies in the GBS cohort. Patients and reactivities against anti-ganglioside antibodies are ordered according to Euclidean clustering. Each row represents one GBS patient. The score of the anti-ganglioside titre is indicated by the colour of the square (0 = < 1/1000, 1 = 1/1000–1/2500, 2 = 1/2500–1/12500, 3 = > 1/12500). Columns in the left contain information related to the clinical variant and the outcome at 6 months and at 1 year of follow-up. [file 12974_2021_2301_MOESM1_ESM.tiff]

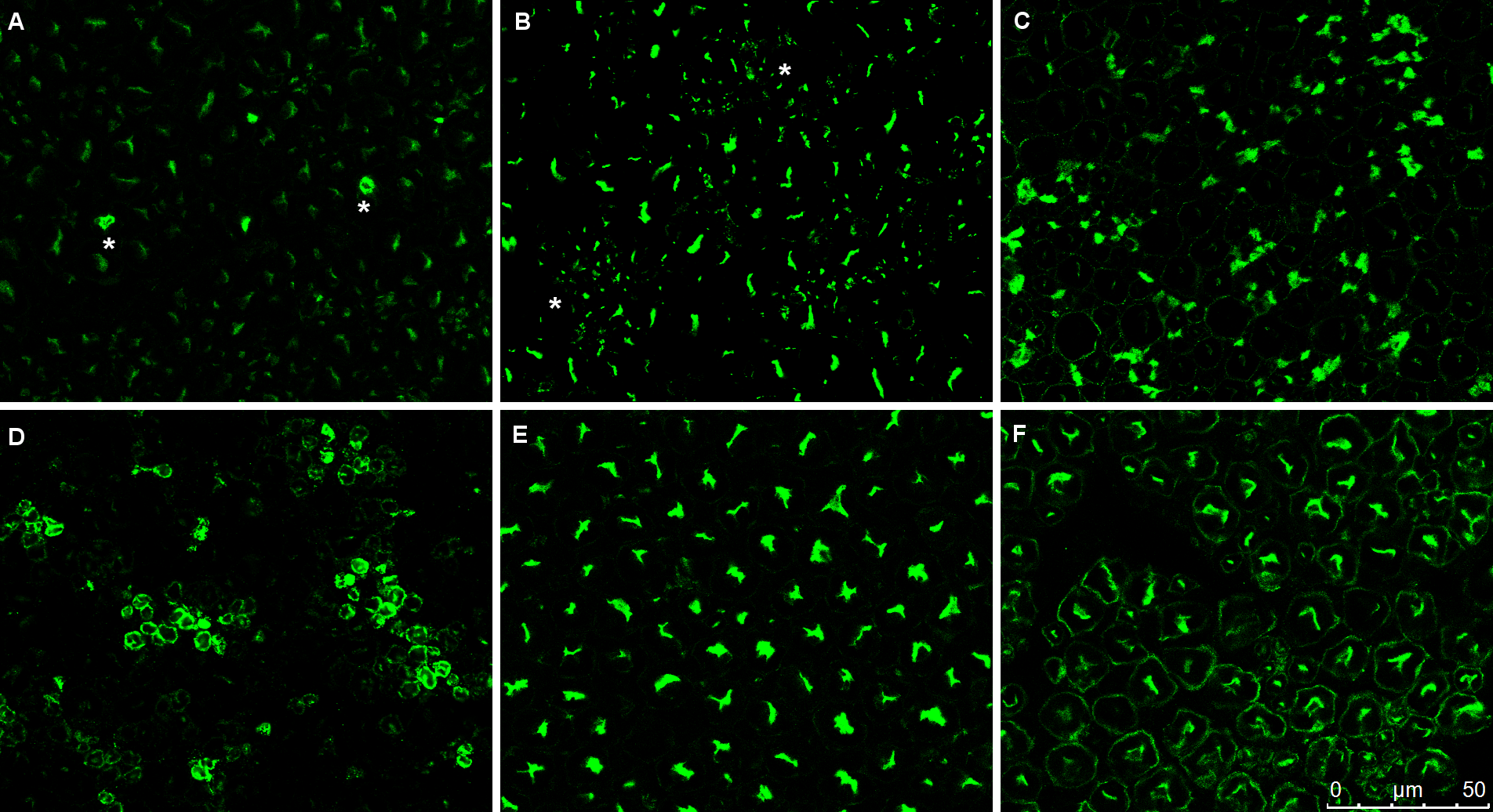

Supplement: Supplementary file 3 — Additional file 3: Figure 3. Staining paterns analized in IHC over monkey peripheral nerve. Macaque peripheral nerve transverse sections stained with CNTN1 positive CIDP patient’s serum reacting against paranodes (A), small fiber axons (B), non-myelinating Schwann cells (C), myelin from small myelinated fibers (D), large fiber axons (E), and myelin from large myelinated fibers (F). [file 12974_2021_2301_MOESM3_ESM.tif]
