# Supplementary material for: Autoantibody screening in Guillain–Barré syndrome
Source: J Neuroinflammation. 2021 Nov 1;18:251. doi: 10.1186/s12974-021-02301-0 (PMC8559393; doi:10.1186/s12974-021-02301-0)
Supplement: Supplementary file 4 — Additional file 4: Table 1. Statistical analysis of structures observed in IHC over monkey peripheral nerve. Table 2. Statistical comparison between GBS patients with and without anti-ganglioside antibodies. [file 12974_2021_2301_MOESM4_ESM.docx]

**Additional information: Additional data**

|  |  | **GBS patients (n =100)** | | **Controls (n=56)** | | **P value** | |
| --- | --- | --- | --- | --- | --- | --- | --- |
|  |  | **Any reactivity** | **Strong reactivity** | **Any reactivity** | **Strong reactivity** | **Any reactivity** | **Strong reactivity** |
| **IgG** | **Nodes/paranodes** | 0 (0%) | 0 (0%) | 0 (0%) | 0 (0%) | >0.9999 | >0.9999 |
|  | **Myelin from small myelinated fibers** | 13 (13%) | 8 (8%) | 2 (3.6%) | 0 (0%) | 0.0866 | 0.0512 |
|  | **Myelin from large myelinated fibers** | 11 (11%) | 7 (7%) | 4 (7.1%) | 0 (0%) | 0.5751 | 0.0501 |
|  | **Schwann cells from unmyelinated fibers** | 15 (15%) | 6 (6%) | 3 (5.4%) | 1 (1.8%) | 0.1147 | 0.4228 |
|  | **Large fiber axons** | 49 (49%) | 4 (4%) | 20 (35.7%) | 2 (3.6%) | 0.1313 | >0.9999 |
|  | **Small fiber axons** | 26 (26%) | 1 (1%) | 21 (37.5%) | 0 (0%) | 0.1484 | >0.9999 |
|  | **All Schwann cells reactivity** | 18 (18%) | 13 (13%) | 7 (12.5%) | 1 (1.8%) | 0.4959 | 0.0192(*) |
| **IgM** | **Nodes/paranodes** | 0 (0%) | 0 (0%) | 0 (0%) | 0 (0%) | >0.9999 | >0.9999 |
|  | **Myelin from small myelinated fibers** | 10 (10%) | 4 (4%) | 3 (5.4%) | 2 (3.6%) | 0.3802 | >0.9999 |
|  | **Myelin from large myelinated fibers** | 9 (9%) | 2 (2%) | 4 (7.1%) | 2 (3.6%) | 0.7716 | 0.6185 |
|  | **Schwann cells from unmyelinated fibers** | 7 (7%) | 4 (4%) | 3 (5.4%) | 1 (1.8%) | >0.9999 | >0.9999 |
|  | **Large fiber axons** | 36 (36%) | 6 (6%) | 17 (30.4%) | 3 (5.4%) | 0.5973 | >0.9999 |
|  | **Small fiber axons** | 11 (11%) | 2 (2%) | 12 (21.4%) | 3 (5.4%) | 0.0997 | 0.3507 |
|  | **All Schwann cells reactivity** | 13 (13%) | 7 (7%) | 6 (10.7%) | 3 (5.4%) | 0.8012 | >0.9999 |

**Additional table 1.** Statistical analysis of structures observed in IHC over monkey peripheral nerve.

Comparison between GBS patients and controls. *Strong reactivity* includes scores 2 and 3, and *any reactivity* includes scores 1, 2 and 3. Fluorescence intensity scores were analysed using contingency analysis with the application of a Fisher’s exact test, accepting an alpha-level of <0.05 to determine significance.

**Additional table 2.** Statistical comparison between GBS patients with and without anti-ganglioside antibodies.

|  | **GBS patients with anti-ganglioside antibodies**  **(n = 61)** | | **GBS patients without anti-ganglioside antibodies (n = 39)** | | **P value** | |
| --- | --- | --- | --- | --- | --- | --- |
|  | **Any reactivity** | **Strong reactivity** | **Any reactivity** | **Strong reactivity** | **Any reactivity** | **Strong reactivity** |
| **Neuroblastoma neurons IgG** | 6 (9.8%) | 2 (3.3%) | 5 (12.8%) | 0 (0%) | 0.747 | 0.519 |
| **Neuroblastoma neurons IgM** | 13 (21.3%) | 3 (4.9%) | 15 (38.5%) | 5 (12.8%) | 0.072 | 0.256 |
| **DRG neurons IgG** | 22 (36.1%) | 5 (8.2%) | 9 (23.1%) | 1 (2.6%) | 0.191 | 0.400 |
| **DRG neurons IgM** | 24 (39.3%) | 8 (13.1%) | 10 (25.6%) | 3 (7.7%) | 0.196 | 0.521 |
| **Monkey peripheral nerve IgG** | 37 (60.7%) | 10 (16.4%) | 19 (48.7%) | 7 (17.9%) | 0.303 | >0.999 |
| **Monkey peripheral nerve IgM** | 22 (36.1%) | 6 (9.8%) | 22 (56.4%) | 6 (15.4%) | 0.063 | 0.530 |
| **Nodes/paranodes IgG** | 0 (0%) | 0 (0%) | 0 (0%) | 0 (0%) | - | - |
| **Myelin from small myelinated fibers IgG** | 7 (11.5%) | 5 (8.2%) | 6 (15.4%) | 3 (7.7%) | 0.561 | >0.999 |
| **Myelin from large myelinated fibers IgG** | 5 (8.2%) | 3 (4.9%) | 6 (15.4%) | 4 (10.3%) | 0.331 | 0.427 |
| **Schwann cells from unmyelinated fibers IgG** | 8 (13.1%) | 4 (6.6%) | 7 (17.9%) | 3 (7.7%) | 0.572 | >0.999 |
| **Large fiber axons IgG** | 32 (52.5%) | 3 (4.9%) | 17 (43.6%) | 1 (2.6%) | 0.418 | >0.999 |
| **Small fiber axons IgG** | 17 (27.9%) | 1 (1.6%) | 9 (23.1%) | 0 (0%) | 0.647 | >0.999 |
| **All Schwann cells reactivity IgG** | 10 (16.4%) | 7 (11.5%) | 8 (20.5%) | 6 (15.4%) | 0.605 | 0.561 |
| **Nodes/paranodes IgM** | 0 (0%) | 0 (0%) | 0 (0%) | 0 (0%) | - | - |
| **Myelin from small myelinated fibers IgM** | 4 (6.6%) | 3 (4.9%) | 6 (15.4%) | 1 (2.6%) | 0.182 | >0.999 |
| **Myelin from large myelinated fibers IgM** | 3 (4.9%) | 1 (1.6%) | 6 (15.4%) | 1 (2.6%) | 0.148 | >0.999 |
| **Schwann cells from unmyelinated fibers IgM** | 3 (4.9%) | 2 (3.3%) | 4 (10.3%) | 2 (5.1%) | 0.427 | 0.642 |
| **Large fiber axons IgM** | 17 (27.9%) | 2 (3.3%) | 19 (48.7%) | 4 (10.3%) | 0.054 | 0.205 |
| **Small fiber axons IgM** | 2 (3.3%) | 0 (0%) | 9 (23.1%) | 2 (5.1%) | 0.003 (**) | 0.150 |
| **All Schwann cells reactivity IgM** | 5 (8.2%) | 4 (6.6%) | 8 (20.5%) | 3 (7.7%) | 0.125 | >0.999 |

Comparison between GBS patients with and without anti-ganglioside antibodies. *Strong reactivity* includes scores 2 and 3, and *any reactivity* includes scores 1, 2 and 3. Fluorescence intensity scores were analysed using contingency analysis with the application of a Fisher’s exact test, accepting an alpha-level of <0.05 to determine significance.
